# Clinicopathological analysis of rosette-forming glioneuronal tumors

**DOI:** 10.1186/s13000-024-01465-6

**Published:** 2024-02-22

**Authors:** Jing Liu, Fan Lin, Yanhua Sun, Xia Liu

**Affiliations:** 1 Department of Pathology, Shenzhen Second People’s Hospital, Shenzhen University 1st Affiliated Hospital, Shenzhen University School of Medicine, Shenzhen, 518035 China; 2 Department of Radiology, Shenzhen Second People’s Hospital, Shenzhen University 1st Affiliated Hospital, Shenzhen University School of Medicine, Shenzhen, 518035 China

**Keywords:** Rosette-forming glioneuronal tumor, Pilocytic astrocytoma, Clinical pathology, Molecular diagnosis

## Abstract

**Background:**

This study aimed to investigate the clinicopathological characteristics, diagnostic indicators, and critical factors for the differential diagnosis of rosette-forming glioneuronal tumor (RGNT).

**Patients and methods:**

This retrospective study included six surgically treated RGNT cases. We analyzed and summarized their clinical manifestations, radiological features, histological morphology, immunophenotype, and molecular genetic changes, supplemented with a literature review.

**Results:**

The patients comprised four males and two females with a mean age of 35 years. The tumors were located in the cerebellum (two cases); the fourth ventricle, quadrigeminal cistern, and third ventricle (one case each); and the fourth ventricle and brainstem (one case). Clinical manifestations included headaches in four cases, left eyelid ptosis in one case, and one asymptomatic case only identified during physical examination. Microscopically, the tumor cells were uniform in size and were marked by rosette-like or pseudorosette-like structures around the neuropil and blood vessels. Immunohistochemistry revealed biphasic patterns. The central neuropil components of the rosette-like structures around the neuropil and the pseudorosette structures of the perivascular regions expressed Syn, while the cells surrounding the rosettes expressed Olig2 and not GFAP. GFAP and S-100 were expressed in the glial components but not in the rosette or pseudorosette regions. The Ki-67 proliferation index was typically low. Molecular genetic analysis showed that the main molecular changes involved *FGFR1* mutation accompanied by *PIK3R1* mutation. None of the patients received chemoradiotherapy postoperatively. Follow-up durations varied between 4 and 23 months with no recorded recurrence or metastasis.

**Conclusion:**

RGNT is a comparatively rare mixed glioneuronal tumor that occurs in the midline structures. Its morphology shows certain overlaps with other low-grade neuroepithelial tumors. Identifying the rosettes around the neuropil is critical for morphological diagnosis, and the molecular identification of *FGFR1* mutations accompanied by *PIK3R1* mutations can facilitate diagnosis.

## Introduction

Rosette-forming glioneuronal tumor (RGNT) is a mixed glioneuronal tumor that consists of two separate histological components. One component is characterized by uniform rosette-like structures around the neuropil or pseudorosette structures in the perivascular regions. The other features glial cell components exhibiting pilocytic or oligodendroglial morphology similar to pilocytic astrocytoma. These tumors are characterized by *FGFR1* mutations, which are frequently accompanied by *PIK3CA* and/or *NF1* mutations [1]. In 2002, Komori et al. first described RGNT as a novel mixed neuroglial tumor entity, and the 4th edition of the World Health Organization (WHO) *classification of central nervous system tumors* (2007) classified RGNT as a distinct entity known as “fourth ventricle rosette-forming glioneuronal tumor” [2, 3]. Further understanding of this tumor has led to successive reports on intracranial space-occupying lesions in the spinal cord, posterior cranial fossa, pineal body, and in cases with multiple lesions. Therefore, in the revised 4th edition of the WHO classification (2016) and 5th edition (2021), the tumor was renamed from “fourth ventricle rosette-forming glioneuronal tumor” to “rosette-forming glioneuronal tumor.” Related molecular pathological features were also incorporated into the classification [4]. 

RGNT generally has a good prognosis, with very few reports of recurrence or progression. Pathologically, it predominantly requires differentiation from pilocytic astrocytoma and diffuse leptomeningeal glioneuronal tumor. Reports analyzing the clinical pathology of this tumor in China are relatively limited. This paper presents an analysis of the clinicopathological characteristics, radiological features, and prognosis of six RGNT cases to add knowledge regarding this tumor.

## Materials and methods

### Data source

Data regarding six cases diagnosed as RGNT between March 2016 and May 2022 were obtained from the Pathology Department of the Shenzhen Second People’s Hospital (First Affiliated Hospital of Shenzhen University). Information such as clinical, radiological, and pathological data were compiled, and light microscopic morphology of the tumor tissues was evaluated. Other information analyzed include patient sex, age, growth location, tumor size, radiological features, immunohistochemical properties, molecular test results, completeness of surgical excision, and follow-up circumstances. Pathological sections were reviewed by an attending physician and a chief physician. All six patients were followed up as outpatient via hospital information system or by phone until August 30, 2022. This study was carried out in accordance with the principles of the Helsinki Declaration and approved by the Ethics Committee of the Shenzhen Second People’s Hospital.

### Methods

All specimens were fixed in 10% neutral buffered formalin, followed by routine dehydration, paraffin embedding, and sectioning at 4 μm thickness. The sections were then stained with hematoxylin and eosin and examined using light microscopy. Immunohistochemical staining was performed following the EnVision two-step procedure. The primary antibodies included BRAF V600E, Syn, p53, IDH1 R132H, CD34, S-100, Ki-67, GFAP, Olig-2, H2K27M, H3K27me3, all of which were procured from Fuzhou Maixin Biotech Co., Ltd. All immunohistochemistry procedures were performed on a Maixin fully automatic immunohistochemical staining instrument, strictly adhering to the instructions in all operational steps.

## Results

### Clinical data (Table 1)

Among the six patients, four were males and two were females aged 27–51 years, with an average age of 35 years. The tumor location varied, located in the cerebellum in two cases, the third ventricle in one case, the quadrigeminal cistern in one case, the fourth ventricle in another case, and both the fourth ventricle and brainstem in one case. The patients had nearly the same symptoms: three reported headaches, one had headaches and dizziness, one had left eyelid ptosis, and one was asymptomatic with the tumor detected only during routine physical examination.

**Table 1:** Clinicopathological characteristics of rosette-forming glioneuronal tumors in six cases

Case	Sex	Age (years)	Location of lesion(s)	Size of lesion (mm)	Main symptoms	Treatment	Follow-up (months)
1	Female	51	Left cerebellum	34×23	Headache	Partial surgical resection	4, survival
2	Male	27	Fourth ventricle	33×26×19	Headache and dizziness	Total surgical removal	21, Tumor-free survival
3	Male	31	Right cerebellum	38×31×21	No special symptoms	Total surgical removal	23, Tumor-free survival
4	Male	32	Brainstem, fourth ventricle	43×31	Left eyelid ptosis	Partial surgical removal	21, survival
5	Male	26	Quadrigeminal cistern	21×18×17	Headache	Total surgical removal	21, Tumor-free survival
6	Female	45	Third ventricle	13×12×12	Headache	Total surgical removal	Lost to follow-up

**Fig. 1:**
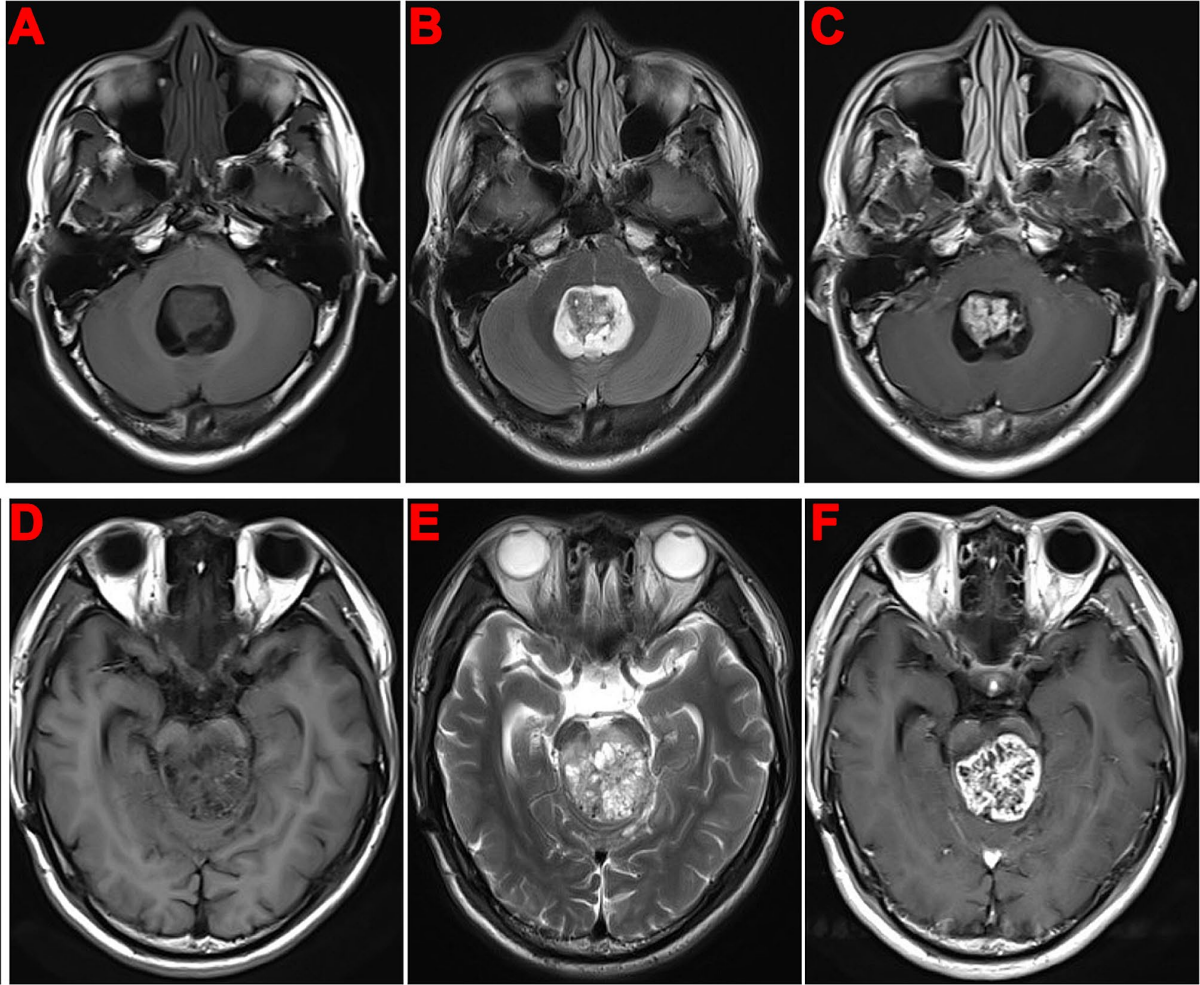
Head MRI: **A-C**, In case 2, the fourth ventricle was enlarged, containing an irregular tumor with an abnormal signal. The tumor displayed mixed medium and low signals on T1WI, with mixed high signals on T2WI. The lesion center appeared as an isointense signal. Contrast-enhanced scanning revealed significant enhancement within the substantial region of the lesion, and slight cystic changes were observed; **D-F**, In case 4, a round space-occupying lesion was identified in the brainstem. T1WI revealed an uneven low signal, and T2WI displayed an uneven high signal. Multiple small cystic changes were observed at the edge of the lesion. On plain scanning, the lesion border was unclear, and mild edema was present. Following enhanced scanning, the lesion demonstrated an uneven enhancement, with the cystic part unenhanced and the lesion border clearer

The duration of the disease ranged from five days to one year. Cranial magnetic resonance imaging (MRI) investigations revealed the following: Case 1 exhibited multiple rounded, cyst-like signal masses with low T1 signal and high T2 signal. Case 2 displayed an irregularly shaped tumor with an abnormal signal, characterized by mixed medium and low T1 signals and high T2 signal (Fig. 1A-C). For Case 3, the lesion was clear with abnormal signals, particularly low T1 and high T2 signals. Case 4 had mixed space-occupying lesions, prolonged T1 and T2 signals, mixed signals, and multiple cystic degenerations (Figures D-F). Case 5 showed abnormal signals, with a patchy high T1 signal and an unevenly high T2 signal. Case 6 showed nodular abnormal signals, a low T1 signal, and a uniformly high T2 signal.

Post-contrast enhancement indicated that four cases showed substantial enhancement, whereas two revealed no enhancement. All six cases underwent surgical resection, with total resection performed in four cases and partial resection conducted in the other two.

### Pathological examination

**Fig. 2:**
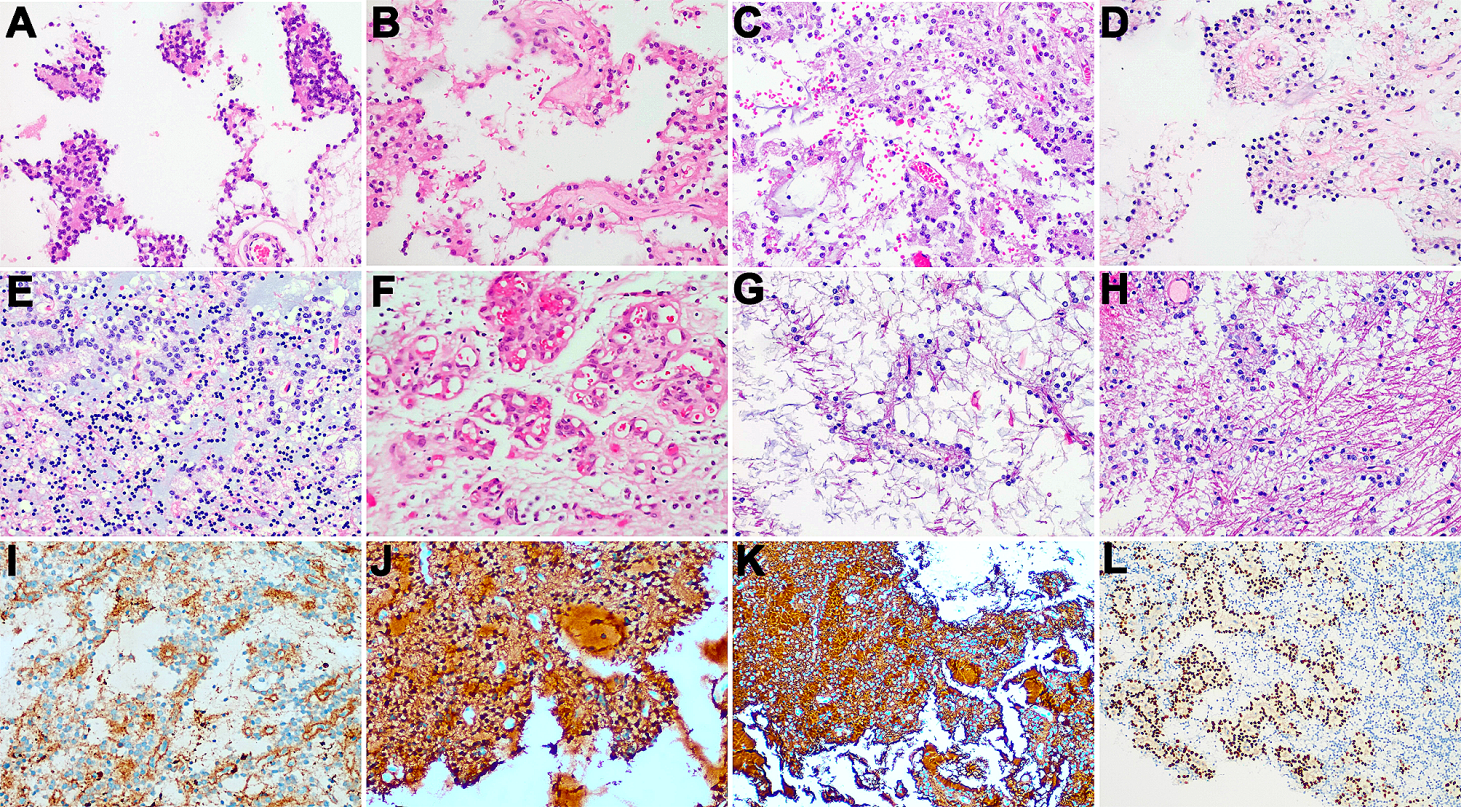
HE staining and immunohistochemical staining: **A**-Perineuronal rosettes or pseudorosettes formed by neurons around the neurofibrillary tangles were observed more clearly or formed around the blood vessels; **E**, The display of a mucoid matrix was similar to an area of dysembryoplastic neuroepithelial tumor (DNET); **F**, Microvascular proliferation was observed; **G-H**, Some areas bore semblance to pilocytic astrocytoma; **I**, The central neuropil components of the rosette-like structures around the neuropil or the pseudorosette structures of the perivascular regions expressed Syn; **J**, S-100 expression was present in the glial components; **K**, GFAP was expressed in the glial components; **L**, The cells around the rosettes were observed to express Olig2 but did not express GFAP. GFAP and S-100 were expressed in the glial components

Pathological examination revealed the following:

1. Gross and microscopic examinations revealed that all tumor tissues subjected to examination were fragmented tissues and exhibited a soft texture with colors ranging from gray-white to gray-red and gray-brown.
2. Under microscopic examination, histological morphologies across the six cases were similar. The boundary between tumor and normal brain tissues was unclear, with tumor cells showing low-to-moderate density, fairly uniform cell size, round nuclei, granular chromatin, inconspicuous nucleoli, and minimal cytoplasm. Cystic areas were noticeable, with no evidence of mitosis or necrosis. Regions resembling oligodendroglioma were observed in all cases, with four cases displaying distinct neuronal formations appearing as rosette-like structures around the neuropil or pseudorosette structures in perivascular regions (Fig. 2A-D). Two cases presented with indiscernible neuronal formations in similar regions. Eosinophilic bodies were observed in cases 1, 2, and 6, while calcification was noted in cases 1 and 3. Cases 1, 2, and 5 exhibited microvascular hyperplasia (Fig. 2F), and case 1 showed dispersed multinucleated cells and calcification. Mucinous matrices similar to regions of dysplastic neuroepithelial tumor (DNET) (Fig. 2E) were observed in cases 3 and 4, and areas similar to pilocytic astrocytoma were identified in cases 5 and 6 (Fig. 2G and H).
3. Immunohistochemistry revealed similar biphasic immune phenotypes across all the tumor cases. The central neuropil components of the rosette-like structures around the neuropil and the pseudorosette structures of the perivascular regions expressed Syn **(**Fig. 2I**)**, while cells around the rosette structures expressed Olig2 **(**Fig. 2L**)** and not GFAP. GFAP and S-100 were expressed in glial components **(**Fig. 2J **and K)** but not in rosettes or pseudorosettes. The remaining immunohistochemical markers (BRAF V600E, IDH1 R132H, CD34, and H2K27M) were all negative except for P53, which showed sporadic weak positivity (wild-type expression). The Ki-67 proliferation index was low (1–3%).
4. Regarding molecular genetics, Sanger sequencing in case 1 revealed wild-type *IDH1* and *IDH2*, while polymerase chain reaction (PCR) indicated negative MGMT methylation. For case 2, Sanger sequencing identified wild-type *IDH1*, *IDH2*, and TERT promoter, with PCR reporting BRAF V600E as wild-type and MGMT methylation as negative. Neither 1p19q chromosome deletion nor BRAF-KIAA1549 fusion was detected via FISH in case 2. Case 3 displayed wild-type *IDH1* and *IDH2* as determined by Sanger sequencing, BRAF V600E as wild-type as determined by PCR, and no 1p19q chromosome deletion as shown in the FISH results. In case 4, Sanger sequencing detected wild-type *IDH1* and *IDH2*. Second-generation sequencing for case 5 revealed *FGFR1* and *PIK3R1* mutations. No molecular detection was performed for case 6.

### Follow-up

None of the six patients received chemoradiotherapy post-surgery. Four months post-surgery, a cranial MRI re-examination of the patient in case 1 revealed no tumor progression. The patient in case 2 underwent cranial MRI re-examinations thrice over 21 months post-surgery, with no recurrence observed. The patient in case 3 was re-examined four times by cranial MRI in the 23 months following surgery, showing no signs of recurrence. In the case 4 patient, a cranial MRI conducted 21 months post-surgery revealed no evidence of tumor progression. Similarly, no recurrence was detected in case 5 upon cranial MRI re-examination 21 months post-surgery. The patient in case 6 was lost to follow-up.

## Discussion

RGNT is an independent class of glioneuronal tumors. Initially reported in 1995 by Kuchelmeister et al., the tumor was identified as a dysembryogenic neuroepithelial tumor. Later, in 2002, Komori et al. classified this group of tumors as a new tumor type, naming it a ‘rosette-forming glioneuronal tumor.’ [2, 5].

It is generally believed that RGNT often occurs in the fourth ventricle, cerebellar vermis, cerebellar hemisphere, and adjacent regions. Instances of tumors found in the spinal cord, thalamus, pons, optic chiasma area, tegmental area, pineal body, and cases with multiple intracranial space-occupying lesions have been documented, with few patients also presenting with type I neurofibromatosis [6–11]. In our study involving six RGNT cases, three were situated in the fourth ventricle or cerebellum, one in the quadrigeminal cistern, one in the third ventricle, and another exhibited multiple space-occupying lesions involving the brainstem, fourth ventricle, cerebellum, and bilateral thalamus. Our findings indicate no substantial difference in clinical prognosis between cases located in the fourth ventricle and cerebellum compared to those in other rare locations. The clinical presentations of RGNT are closely related to the lesion location, with associated symptoms including headaches, cerebellar ataxia, vision impairment, heavy vomiting, and dizziness [12, 13]. Among the six cases in our study, three presented with headaches, one presented with headaches and dizziness, one presented with left eyelid ptosis, and one was asymptomatic with mass detected during physical examination.

Typically, RGNT exhibits well-defined boundaries in imaging, appearing as cystic, compact cystic, or multicystic space-occupying lesions. CT imaging reveals a low-density shadow, while MRI demonstrates low signal or isointense signal on T1WI and high signal on T2WI. Given the tumor’s low malignancy potential and sparse cell distribution, DWI typically does not exhibit significant diffusion restriction. Gao et al. suggested that the “green pepper sign” is a characteristic enhancement of RGNT, whereby a diaphragm-like structure, resembling the cross-section of green pepper, appears in the solid central part of the lesion. This region is enhanced in the enhanced scan except for the peripheral cystic part [14]. 

Microscopically, RGNTs present with a distinctive bi-directional differentiation comprising neurocytic and glial components. The neurocytic component comprises uniformly shaped neurocytes creating rosette-like structures around the neuropil or the pseudorosette structures of the perivascular regions. A hallmark of the neurocytic rosettes is the ring-like arrangement of neurocyte nuclei encircling the eosinophilic neuropil. For pseudorosettes, the defining feature is uniformly sized neurocytes arrayed in a radial pattern around blood vessels. In longitudinal observations, both morphological structures may manifest as columnar arrangements of neurocytes. These formations may exist within microcysts or mucinous matrices. The glial component of RGNTs generally dominates and frequently resembles pilocytic astrocytomas in many areas. Astrocytoma cells are spindle- or star-shaped cells, with elongated or oval nuclei and medium-density chromatin. These cells frequently form a fibrous background, varying from dense to loose. In certain areas, the glial component may undergo microcystic changes, forming round or oval shapes, with oligodendrocyte-like cells featuring a halo around the nuclei. Deposits of Rosenthal fibers and eosinophilic granular bodies can be found. Blood vessels may appear dilated, thin-walled, or hyalinized vessels. Thrombus formation or glomeruloid vessels can occasionally be observed. Moreover, ganglion-like cells might be sporadically noticed, although no developmental abnormalities have been observed in the cortex adjacent to the lesion [2, 15]. 

Immunohistochemical Syn expression associates with the centers of neurocytic rosettes and the neuropil of pseudorosettes. In some cases, NeuN can be expressed in neurocytic tumor cells. Olig2 is usually expressed in the nuclei of tumor cells. GFAP and S-100 are expressed in the glial component but not in the rosette and pseudorosette components [2, 15]. 

Genetically, the primary interactions of RGNTs involve the MAPK and PI3K pathways. The continuous activation of the FGFR signaling pathway and frequent *PIK3CA* mutations might drive the formation of these tumors [16, 17]. RGNTs have been discovered in patients with neurofibromatosis type 1 or Noonan syndrome [18, 19]. Epigenetically, RGNTs exhibit a specific methylation expression profile [17]. Genomically, hotspot mutations in *FGFR1* are characteristic, with most cases also displaying *PIK3CA* mutations and a subset of cases showing loss-of-function *NF1* mutations.

RGNTs primarily need to be differentiated from the following tumors:

1. Pilocytic astrocytoma: these often manifest in the cerebellum, with a biphasic characteristic histological features, comprised of dense regions of bipolar cells containing Rosenthal fibers and loose regions of multipolar cells exhibiting microcysts and eosinophilic granular bodies. These tumors may display large nuclei, degenerative atypia, and giant cell tumors. On the genetic front, they typically feature a BRAF-KIAA1549 fusion or BRAF V600E mutation. Although RGNTs are usually present in the fourth ventricle, they can also be found in the cerebellum. The regions of glial cells frequently demonstrate characteristics of pilocytic astrocytoma; however, pilocytic astrocytoma does not present with neuronal cell rosettes or perivascular pseudorosettes. In cases with atypical morphology, the molecular detection of *FGFR1* mutation accompanied by *PIK3CA* mutation can provide further support for an RGNT diagnosis.
2. Diffuse leptomeningeal glioneuronal tumors: these cells proliferate extensively within the leptomeninges or grow in small nests and is often accompanied by mucoid degeneration. Minor portion of the tumor exhibits strong neuronal differentiation, manifesting as neuronal rosettes, perivascular pseudorosettes, or neurocytic islands or displaying a ganglion cell morphology. The parenchymal components may display a DNET-like morphology or an oligodendroglioma-like appearance. The specific location of the disease site in the leptomeninges can aid in differentiating it from RGNT. Moreover, characteristic molecular changes include a deletion in chromosome 1p and alterations in MAPK pathway genes, typically the fusion of KIAA1549 and BRAF.
3. Dysplastic neuroepithelial tumors: these are typically seen in the temporal lobe and are morphologically characterized by a multinodular intracortical growth pattern, with oligodendroglial-like cells arranged in columns. Between these columns, histologically normal neurons appear to be floating in a mucinous matrix (a “one frog in one pond” pattern). Occasionally, RGNTs present with DNET-like morphology, in which case tumor location and identifying neuronal rosettes are key to the differential diagnosis. DNETs also frequently exhibit *FGFR1* mutations, usually not accompanied by *PIK3CA* mutations, and a few may harbor *PDGFRA* or *NF1* mutations.
4. Papillary glioneuronal tumors (PGNTs): these usually occur in the supratentorial region in the temporal lobe. Histologically, they present with a bidirectional pattern with glial pseudopapillary structures and inter-papillary components, which can significantly vary in size between cases. Cuboidal tumor cells cover the hyalinized vessels, with monomorphic neurocytes or medium-sized neurons dispersed within the neuroglial background. In this instance, differentiation from RGNT perivascular pseudorosette structures, combined with the lesion site, can assist in the diagnosis. PGNTs may show focal expression of CD34, and molecular change in PRKCA: SLC44A1 fusion.

Similar to other low-grade neuroepithelial tumors, RGNTs display indolent biological behavior and benign clinical course (WHO grade 1) although about half of the cases present with disabling postoperative defects. Most patients do not relapse post-surgery, and in very few cases, reports of tumor spread, recurrence, or progression have been reported.

## Conclusion

In summary, RGNT is a comparatively rare, low-grade mixed glioneuronal tumor that occurs in the midline structures, particularly the fourth ventricle. Its morphology shows certain overlaps with other low-grade neuroepithelial tumors. When regions resembling pilocytic astrocytoma are present, differentiation from pilocytic astrocytoma can be challenging. Identifying the rosettes around the neuropil is critical for morphological diagnosis, and molecular identification of *FGFR1* mutations accompanied by *PIK3R1* mutations can aid in the diagnosis.

## Abbreviations

RGNT: Rosette-forming glioneuronal tumor
DNET: Dysplastic neuroepithelial tumor

## References

[1] Ellezam B, Theeler BJ, Luthra R, Adesina AM, Aldape KD, Gilbert MR (2012). Recurrent PIK3CA mutations in rosette-forming glioneuronal tumor. Acta Neuropathol.

[2] Komori T, Scheithauer BW, Hirose T (2002). A rosette-forming glioneuronal tumor of the fourth ventricle: infratentorial form of dysembryoplastic neuroepithelial tumor??. Am J Surg Pathol.

[3] Louis DN, Ohgaki H, Wiestler OD, Cavenee WK, Burger PC, Jouvet A (2007). The 2007 WHO classification of tumours of the central nervous system. Acta Neuropathol.

[4] Louis DN, Perry A, Wesseling P (2021). The 2021 WHO classification of tumours of the central nervous system: a summary. Acta Neuropathol.

[5] Kuchelmeister K, Demirel T, Schlörer E, Bergmann M, Gullotta F (1995). Dysembryoplastic neuroepithelial tumour of the cerebellum. Acta Neuropathol.

[6] Duan L, Zhang YK, Fu W, Geng S (2017).

[7] Cebula H, Chibbaro S, Santin MN, Kremer S, Chaussemy D, Proust F (2016). Thalamic rosette-forming a glioneuronal tumor in an elderly patient: case report and literature review. Neurochirurgie.

[8] Tanaka F, Matsukawa M, Kogue R, Umino M, Maeda M, Uchida K (2019). A case of a rosette-forming glioneuronal tumor arising from the pons with disappearance of contrast enhancement. Radiol Case Rep.

[9] Sekar A, Rudrappa S, Gopal S, Ghosal N, Rai A (2019). Rosette-forming glioneuronal tumor in opticochiasmatic region-novel entity in new location. World Neurosurg.

[10] Sieg EP, Payne R, Langan S, Specht CS (2016). Case report: a rosette-forming glioneuronal tumor in the tectal plate in a patient with neurofibromatosis type I. Cureus.

[11] Muhammad S, Hafez A, Karppinen A, Niemelä M (2020). Surgical treatment of a rare rosette-forming glioneuronal tumor in the pineal region. Surg Neurol Int.

[12] Wilson CP, Chakraborty AR, Pelargos PE, Shi HH, Milton CK, Sung S (2020). Rosette-forming glioneuronal tumor: an illustrative case and a systematic review. Neurooncol Adv.

[13] Yang C, Fang J, Li G, Li S, Ha T, Wang J (2017). Histopathological, molecular, clinical and radiological characterization of rosette-forming glioneuronal tumor in the central nervous system. Oncotarget.

[14] Gao L, Han F, Jin Y, Xiong J, Lv Y, Yao Z (2018). Imaging features of rosette-forming glioneuronal tumours. Clin Radiol.

[15] Preusser M, Dietrich W, Czech T, Prayer D, Budka H, Hainfellner JA (2003). Rosette-forming glioneuronal tumor of the fourth ventricle. Acta Neuropathol.

[16] Lin FY, Bergstrom K, Person R, Bavle A, Ballester LY, Scollon S (2016). Integrated tumor and germline whole-exome sequencing indentifies mutations in MAPK and PI3K pathway genes in an adolescent with rosette-forming glioneuronal tumor of the fourth ventricle. Cold Spring Harb Mol Case Stud.

[17] Sievers P, Appay R, Schrimpf D, Stichel D, Reuss DE, Wefers AK (2019). Rosette-forming glioneuronal tumors share a distinct DNA methylation profile and mutations of PIK3CA and NF1. Acta Neuropathol.

[18] Kemp S, Achan A, Ng T, Dexter MA (2012). Rosette-forming glioneuronal tumour of the lateral ventricle in patient with neurofibromatosis 1. J Clin Neurosci.

[19] Karafin M, Jallo GI, Ayars M, Eberhart CG, Rodriguez FJ (2011). Rosette forming glioneuronal tumor in association with Noonan syndrome: pathobiological implications. Clin Neuropathol.

